# Paracrine Influence of Masquelet's Induced Membrane on Marrow Derived Mesenchymal Stem Cells

**DOI:** 10.1002/jor.26053

**Published:** 2025-02-08

**Authors:** Meredyth Bowman, Gracie Sclamberg, Emma Wessels, Kyle Cragg, Alexis Donneys, Kurt D. Hankenson, Mark E. Hake, Andrea I. Alford

**Affiliations:** ^1^ Department of Orthopedic Surgery University of Michigan School of Medicine, A. Alfred Taubman Biomedical Sciences Research Building Ann Arbor Michigan USA; ^2^ Trinity IHA Medical Group Orthopedics‐Ann Arbor Campus Yspilanti Yspilant Michigan USA

**Keywords:** bone graft, coculture, macrophage, Masquelet's induced membrane technique, MSC

## Abstract

Masquelet's induced membrane technique (MIMT) is a staged surgical procedure that leverages the foreign body induced membrane (IM) that forms around a cement spacer placed into a segmental bone defect to support subsequent bone grafting. The mechanisms by which the IM supports bone consolidation are not fully understood. We present an indirect coculture system for studying IM‐MSC interactions using a rat model of MIMT. Compared to control cells, MSC *Tnap* (alkaline phosphatase) was induced by 4‐ but not 8‐week IM. MSC *Spp1* (Osteopontin) was attenuated by both 4‐ and 8‐week IM. Although *Tnfrsf11b* (osteoprotegrin) in MSC exposed to IM was not different from control cells, it was induced by 8‐week IM compared to 4‐week IM. MSC *Tnfsf11* (RANKL) was reduced by 4‐week and 8‐week IM. MSC *Thbs2* (Tsp2) was induced by 8‐week but not 4‐week IM. Ablation of macrophages in IM blocked the induction of *Thbs2* by 8‐week IM. MSC Col1a1 expression was not affected under any condition tested. TMT proteomics analysis of IM‐conditioned medium revealed 150 unique secreted proteins, 7 of which were differentially abundant (fold change ≥ 2 and FDR corrected *p* ≤ 0.05) in 8‐week versus 4‐week IM secretomes. All differentially abundant proteins were elevated in medium conditioned by 8‐week IM. Our data suggest that factor(s) secreted by IM resident cells affect MSC gene expression, and that duration of IM development influences the potency and nature of this paracrine effect. Patient‐specific factors including age and interval between MIMT surgeries may affect IM biological potency and graft to bone consolidation.

## 1 Introduction

Masquelet's induced membrane technique (MIMT) is a staged surgical procedure that leverages the foreign body induced membrane (IM) that forms around a cement spacer when it is placed into a segmental bone defect ([1, 2] reviewed in reference [3]). At the first surgery, the defect is debrided, and a cement spacer is placed. At the second surgery, the IM is opened, the spacer is removed, the pocket is filled with autologous bone graft, and the IM is repaired. IMs allowed to develop 4–6 weeks in vivo had higher osteogenic and vasculogenic growth factor content compared to IMs developed for longer intervals [4, 5, 6, 7]. However, in practice the IM is allowed to develop for as long as is medically necessary, and clinical series suggest that the interval between surgeries has minimal impact on outcomes [8, 9]. On the other hand, MIMT failure has also been associated with inadequate IM formation [10]. Whether cellular constituents of the IM promote graft to bone consolidation or whether the IM only provides a physical barrier that protects graft is still controversial.

The IM is comprised of two layers: an inner cellular layer directly in contact with the spacer and a thin ECM‐rich layer with highly aligned fibers that corresponds to the fibrous capsule. An additional vascularized loose connective tissue layer containing immune cell infiltrate with macrophage lineage cells and osteoclasts are located adjacent to the fibrous layer [6, 11, 12, 13].

Osteochondral progenitor cells are also present in the IM [5, 14] and potentially contribute to graft to bone consolidation after the second MIMT surgery.

Published bulk RNA sequencing of IM tissue generated in a rat model of MIMT suggest that osteoblast and macrophage lineage cells are the predominate cell types in IM [15]. We hypothesize that these IM‐resident cells make paracrine contributions to progenitor cell differentiation and graft to bone consolidation in the second phase of MIMT. In particular, macrophage lineage cells support normal intramembraneous and endochondral fracture healing [16, 17, 18] and have context specific effects on MSC and osteoblasts (reviewed in [19]). Whether macrophages support graft to bone consolidation during MIMT is not known.

Here, we present a coculture system for studying IM‐MSC interactions in vitro and for probing possible contributions of IM‐resident macrophages. Our results suggest that factors secreted by IM‐resident cells influence MSC gene expression in an IM‐age dependent manner and that IM‐resident macrophages are partially responsible. Proteomic analysis of IM conditioned medium suggests that secretomes of 4‐week and 8‐week IM are of similar composition with the abundance of only a handful of secreted factors increasing substantially during this 1‐month interval. Consistent with the development of a fibrous IM, the elevated secreted factors were primarily ECM proteins.

## 2 Methods

### 2.1 Animals

All animal procedures were conducted at the University of Michigan under IACUC approval and complied with NIH guidelines. Male Sprague Dawley rats were purchased from Envigo. Animals were housed in pairs under specific pathogen free conditions with a 12‐h light/dark cycle and free access to chow and water. Animals had access to enrichment, with the exception that climbing implements were replaced with nestlets after surgery to prevent injury.

### 2.2 Primary Marrow Derived MSC Culture

16‐week‐old male Sprague Dawley rats (*N* = 2) were utilized to obtain a single population of primary MSC for the coculture experiments. After euthanasia, femora and tibiae were dissected and cleaned of soft tissue. The epiphyses were crushed with rongeurs, and the marrow was collected by flushing with PBS. A single cell suspension was obtained using a 23 G needle and a 70‐micron cell strainer. Cells were resuspended in MSC growth medium (αMEM + glutamax (Invitrogen) containing 10% FBS (Fisher Scientific), 100 IU/ml penicillin, and 100 ug/ml streptomycin) and seeded onto a T75 flask. One‐third of the medium was replaced every 3 days until the cells reached confluence. Adherent cells were passaged twice more before freezing at 1 × 10^6^ cells per ml in αMEM containing 30% FBS and 5% DMSO.

### 2.3 Masquelet Surgeries

MIMT was performed on 465 ± 9 gram, 16‐week‐old (*N* = 14) male Sprague Dawley rats as previously described with minor modifications [15]. After anesthesia was obtained with isoflurane, the femur was stabilized by placing a polycarbonate internal fixation plate on the anterolateral aspect of the femur. The plate contained a 5 mm notch which was used as a guide to create an osteotomy with a piezoelectric saw. Pre‐formed PMMA spacers were placed into the defect and secured with a screw. Buprenorphine (0.05 mg/kg) was given pre‐operatively and at 8 h post‐surgery. Carprofen was administered immediately postoperatively and again 24 h later. Animals were monitored twice daily for 72 h and then daily for the 4‐ or 8‐week IM development periods. Surgeries were conducted on 5 animals per day, and each day they were randomly allocated into 4‐week and 8‐week groups. Faxitron radiography was performed immediately after surgery and then once per week for the duration of the study to monitor integrity of fixation. Ex vivo microCT was utilized to visualize the defect and fixation construct at weeks 4 and 8. Fixation was lost on one femur and this animal was replaced to maintain 14 total surgical rats.

After 4 or 8 weeks, animals were euthanized, femurs were removed en bloc, IM tissue was dissected off the medial side of spacer and divided into 4–6 pieces. Femurs with the spacer, fixation device, and remaining IM tissue in place were fixed and decalcified for histology. Dissected IM tissue was divided into pieces and placed into indirect coculture with primary rat MSC as detailed in the following sections.

### 2.4 IM‐MSC Coculture

Approximately 1 week before IM harvest, primary MSC were thawed and seeded onto T75 flasks in MSC growth medium. On the day of IM harvest, MSC were passaged onto 24 well plates at 50,000 cells per well in MSC growth medium. IMs were dissected as described above and incubated in 96 well plates with clodronate liposomes (2.5 ug/ul) or with PBS. The next day, MSC growth medium was replaced with 0.70 ml serum‐free growth medium, and transwell inserts (0.4‐micron filter) containing 0.35 ml serum‐free growth medium were placed into the culture wells. IM samples were then transferred into the transwell inserts. MSC cultured alone in serum‐free growth medium served as baseline controls. 72 h later, IMs were removed and stored at −80°C until further analysis. MSC were photographed with a Lionheart automated microscope and then lysed in Trizol for RNA extraction and gene expression analysis. IM cultured alone in serum‐free growth medium were used to verify reduction of *ITGAM* (cd11b) after chlodronate treatment and to generate conditioned medium from untreated IMs for TMT proteomics.

### 2.5 Determination of IM DNA Content

IMs were thawed, centrifuged at 8000 rpm for 5 min to remove surface water, and wet weights were determined. IMs were then lysed overnight at 55°C in 500 μL of DNA lysis buffer containing 100 mM Tris, 5 mM EDTA, 0.2% SDS, 200 mM NaCl and 5 μL proteinase K. Next, the tubes were vortexed and centrifuged for 10 min at 12,000 rpm. The supernatant was transferred to a new tube and DNA was precipitated with isopropanol. After centrifugation at 12,000 rpm for 15 min, the supernatant was removed, pellets were air dried for 20 min, and then resuspended in 30 μL nuclease‐free water. Samples were incubated at 55°C for 30 min to completely dissolve the DNA. DNA content was determined by UV spectrophotometry.

### 2.6 Gene Expression Analysis

200 ng RNA was reverse‐transcribed in a C1000 thermal cycler (Biorad) with qScript cDNA supermix (Quanta Biosciences). Real time PCR was performed with SyberGreen supermix (Invitrogen) on a CFX96 system (Biorad). MSC genes examined were *TNAP* (alkaline phosphatase), *colIα1*, *thbs2, Spp1*, (osteopontin), *Tnfrsf11b*, (osteoprotegerin), *Tnfsf11* (receptor activator of nuclear factor kappa beta ligand (RANKL)) The macrophage marker *ITGAM* (Cd11b) was measured in IM tissue. Expression levels were normalized to *ATP5b*. Primers were designed at exon‐exon junctions in NCBI Primer Blast using RefSeq mRNA sequences (Table 1).

**Table 1:** PCR primer sequences.

Gene name	Forward	Reverse
TNAP (alk phos)	TCCTTAGGGCCACCGCT	GCGTTGGTGTTGTACGTCTTG
ColIα1	GGAGAGAGCATGACCGATGG	AAGTTCCGGTGTGACTCGTG
Thbs2	ACAACCAAGACAACTGCCCA	ACTCATCGAAACCTACGGCG
Spp1 (osteopontin)	CAGAGGAGAAGGCGCATTACA	AATCCTCGCTCTCTGCATGG
TNFRSF11B (OPG)	GAATGTGAGGAAGGGCGCTA	GCACAGGGTGACATCTATTCC
Tnfsf11 (RANKL)	GCCAACATCCCATCGGGTTC	CCAGTTCTTAGTGCTCCCCC
Integrin αM (cd11b)	GGAAACGCCTTCCACAAACC	CAGCAAGGGACCGTTAGAGG
ATP5b	ATGTTGAGTCTTGTGGGGCG	CGCATAGTCTCTGGCAGGAT

### 2.7 Gross Histology

After decalcification in 0.5 M EDTA, pH 7.6 bones with the hardware and cement spacers intact were embedded in methylmethacrylate plastic. Longitudinal 5‐μm sections were made starting from the medial aspect of the femur. Sections were stained with Masson's trichrome.

### 2.8 Proteomics

Medium collected from 4‐week and 8‐week IM cultured alone was used for analysis at the Proteomics Research Facility at the University of Michigan Department of Pathology. Medium from 2 to 3 biological replicates was combined to achieve 3 technical replicates per group. After trypsin digestion, proteins were multiplexed using the TMT‐10plex mass tag labeling kit (ThermoFisher) and fractionated using 2D liquid chromatography. Orbitrap fusion mass spec was used to obtain peptide sequences. Proteins were identified using Proteome Discoverer v2.1. Valid spectra were identified using the Percolator algorithm in the Proteome Discover package. 150 unique proteins met our selection criteria of ≥ 2 peptide spectral matches and FDR ≤ 5%.

### 2.9 Statistics

IM wet weight and DNA content were compared by linear regression. Differences in gene expression were detected using ANOVA and Tukey post‐hoc tests. *p* < 0.05 was considered significant. For proteomics, normalized abundance values were obtained against the total peptide signal. 8‐week IM conditioned medium values were compared to the 4‐week samples. Proteins were considered differentially abundant at ≥ 2 fold and FDR corrected *p* ≤ 0.05. Graph Pad Prism v10 was used for analysis.

## 3 Results

**Figure 1:**
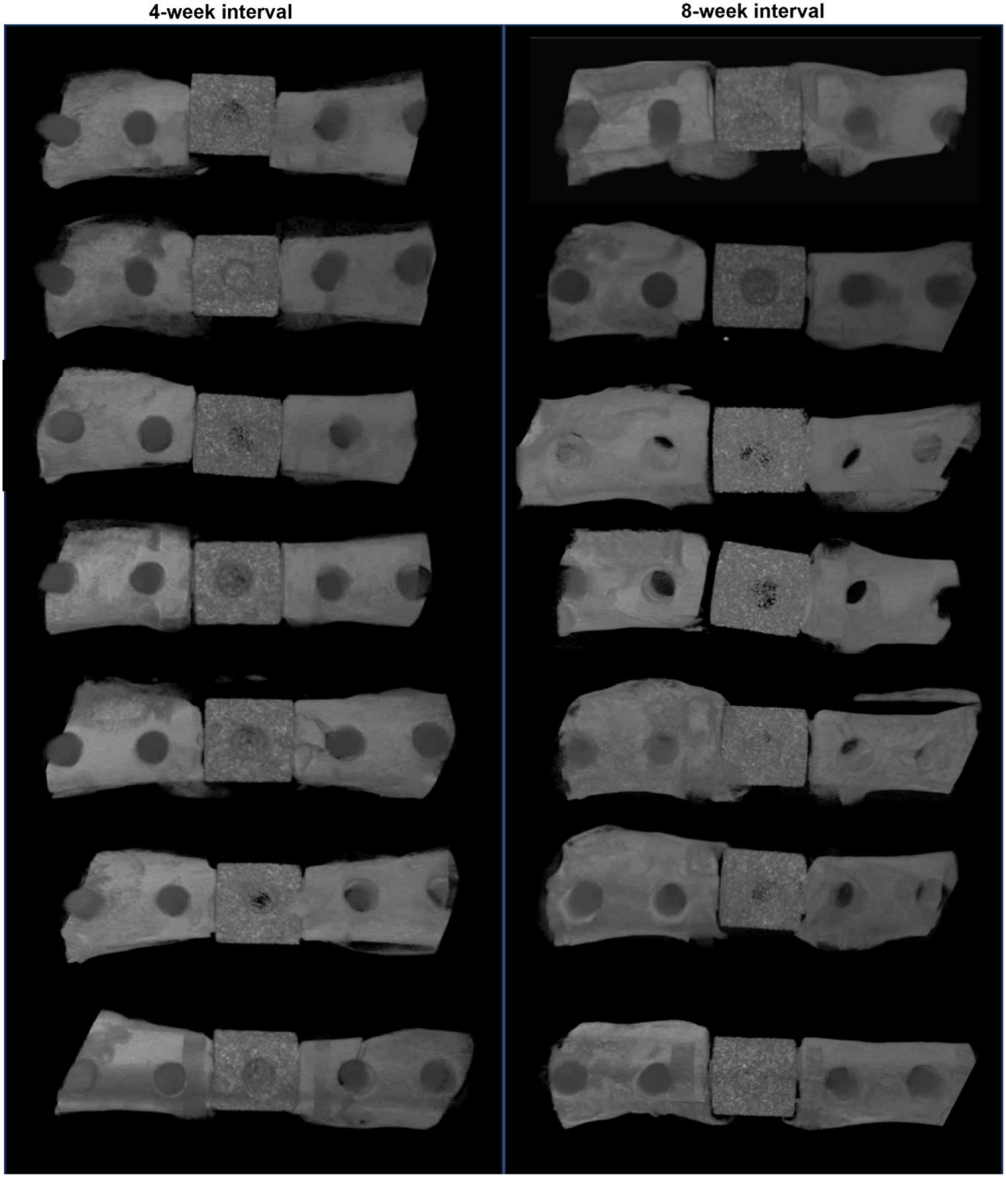
In vivo microCT images obtained the day before sacrifice and dissection. Induced membrane development was allowed to proceed for 4 weeks (left; *N* = 7) or 8 weeks (right; *N* = 7) after MIMT surgery.

In vivo microCT images obtained the day before sacrifice demonstrate that the spacer and fixation construct remained in place for all animals (Figure 1).

**Figure 2:**
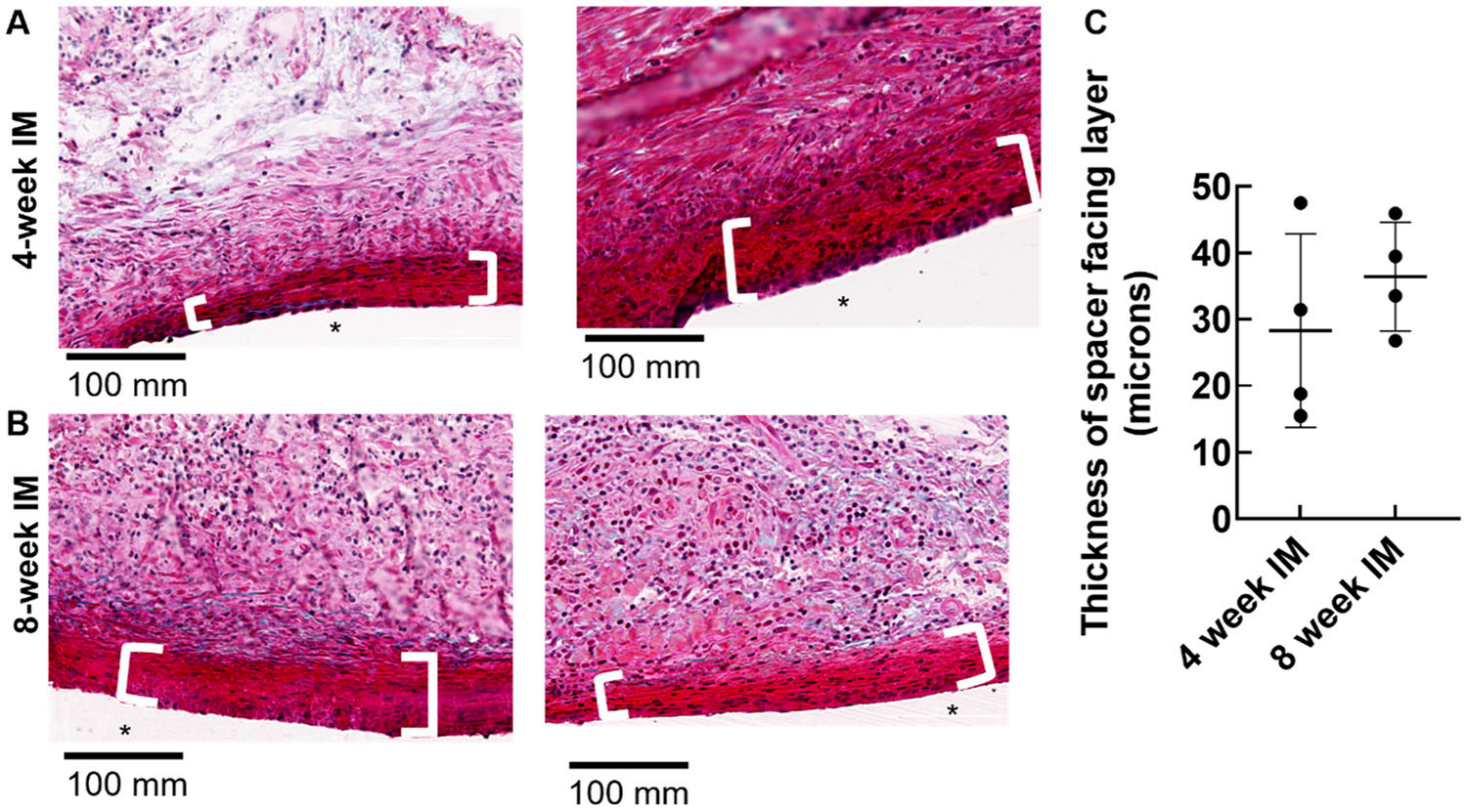
Masson's trichrome histology of IM at the anterior‐lateral facing side of the spacer. After removing IM tissue from the medial face of the spacer, bones were processed for histology (*N* = 4 per IM development interval). At both 4 (A) and 8 (B) weeks, the IM was composed of a variably thick fibrous layer (denoted with with bars and quantified in [C]) adjacent to the spacer plus a second layer containing immune cell infiltrate and blood vessels. *PMMA spacer.

As previously documented by several groups (e.g [20]. and reviewed in [3]), gross histology revealed a thin fibrous layer adjacent to the spacer and a second loose connective tissue layer containing immune cells and blood vessels. Both layers appeared to become denser and more consolidated with time (Figure 2A,B) However, the thickness of the thin fibrous layer was highly variable from animal to animal and around each spacer, and so its thickness was not related to duration of IM development (Figure 2C). We did not measure thickness of the loose connective tissue layer because in several samples, the overlying muscle was lost during plastic processing, thereby preventing confident demarcation of the IM‐muscle boundary.

**Figure 3:**
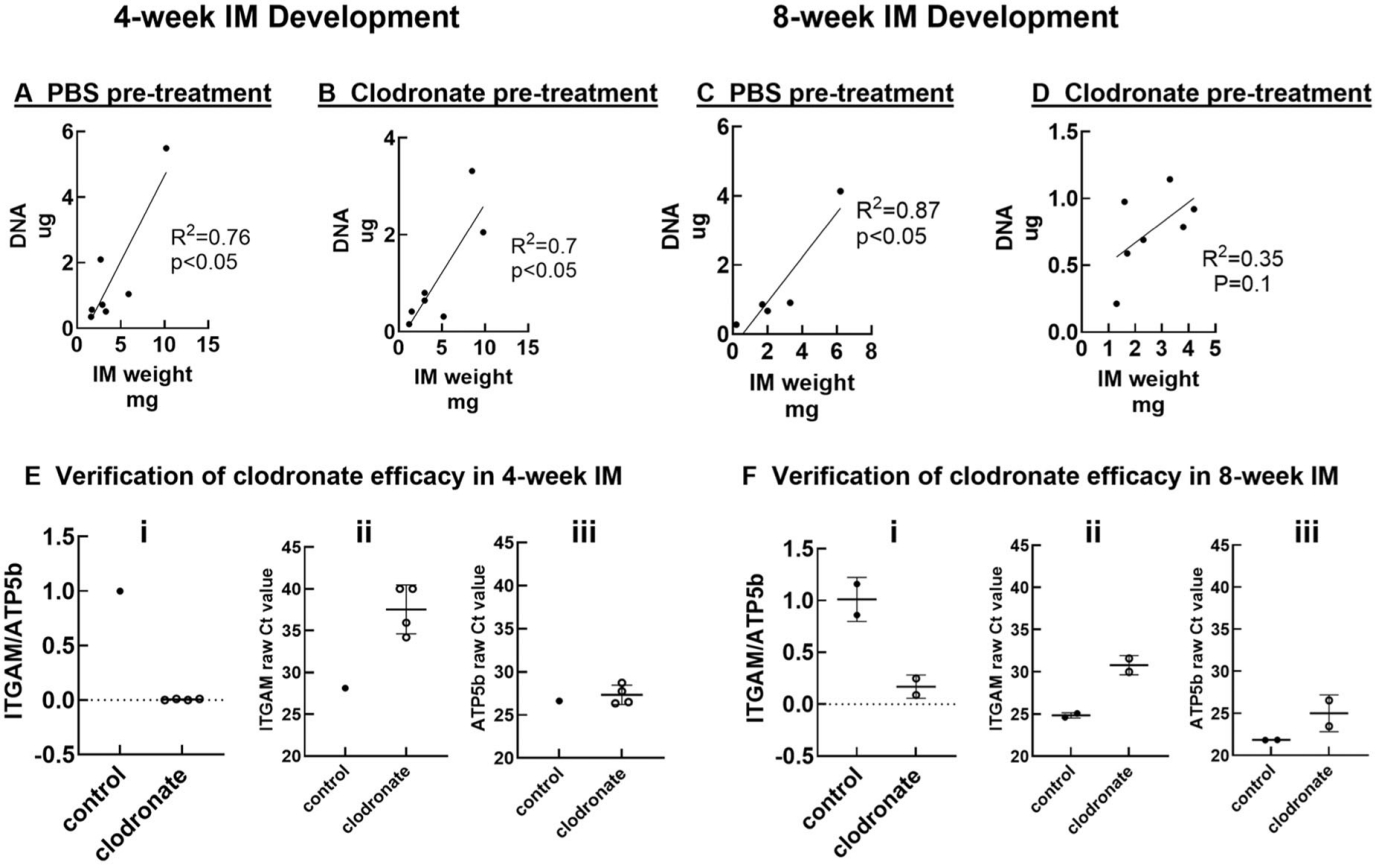
IM attributes after clodronate pretreatment and coculture with MSC. (A–D): Wet weight and DNA content of 4‐week and 8‐week IM were determined after pretreatment with PBS (A, C) or clodronate (B, D), followed by 72 h coculture with MSC. (E and F) Expression of the macrophage surface marker CD11b (integrin alpha M) was reduced in 4‐week (Ei) and 8‐week (Fi) IM treated with clodronate. Raw Ct values (Eii, Eiii, Fii, Fiii) verify that the housekeeping gene was minimally affected by clodronate treatment. Each data point represents IM tissue harvested from a single animal. Data are from 5 to 7 animals per outcome measure.

Published bulk RNA sequencing of IM tissue suggests that osteoblast and macrophage lineage cells are both present in IM. To elucidate possible macrophage‐dependent paracrine effects of IM on MSC phenotype, IMs were treated overnight with clodronate liposomes or PBS, and then placed into indirect coculture with rat primary marrow derived MSC. After 72‐h of coculture, IM weights and DNA content were determined. IM weight and DNA content were positively correlated (*R* ^2^ = 0.76 and 0.87 in 4‐week and 8‐week IM, respectively (Figure 3A,C). Clodronate reduced this correlation in 8‐week (*R* ^2^ = 0.35) but not 4‐week (*R* ^2^ = 0.7) IM (Figure 3B,D). *ITGAM* which encodes the macrophage surface marker CD11b was reduced in the clodronate treatment groups (Figure 3E,F).

**Figure 4:**
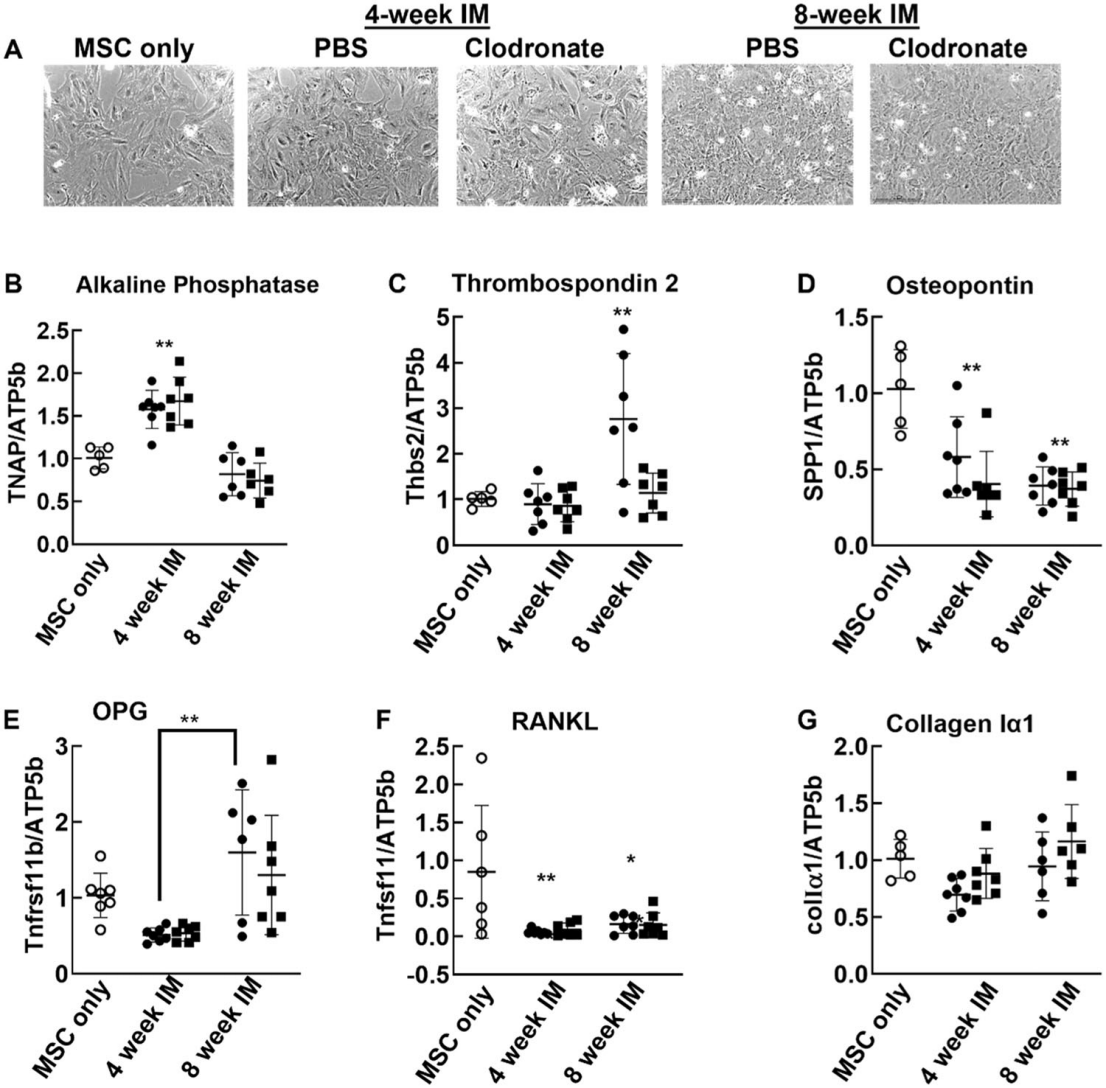
Paracrine effects of IM on primary MSC. After 72 h of indirect coculture with 4‐ or 8‐week IM pretreated with PBS or clodronate, the MSC were photographed (A) and expression of osteoblastic genes were determined in MSC. Gene expression levels were normalized to MSC cultured alone without IM (B–G). Open circles: MSC only. Closed circles MSC cocultured with IM pretreated with PBS. Closed squares: MSC cocultured with IM pretreated with clodronate. *p < 0.05, **p < 0.01 versus MSC or between IM ages as indicated by lines between groups. IM tissue from *N* = 7 rats per IM development interval was subject to coculture. Each data point represents a single culture well.

Next, MSC that had been in coculture were photographed with a Lionheart automated microscope (Figure 4A) and then processed for rtPCR. *Tnap* (alkaline phosphatase) was induced in MSC cocultured with 4‐week (*p* < 0.01), but not 8‐week IM (Figure 4B). *Thbs2* (thrombospondin 2 [TSP2]) was induced in MSC cocultured with 8‐week (*p* < 0.01) but not 4‐week IM. Clodronate pretreatment eliminated the induction of *Thbs2* by 8‐week IM (Figure 4C). *Spp1* (osteopontin) was attenuated in MSC cocultured with both 4‐(*p* < 0.01) and 8‐(*p* < 0.001) week IM (Figure 4D). Although *Tnfrsf11b* (osteoprotegrin [OPG]) in MSC exposed to IM was not different from control, it was higher in cells exposed to 8‐week compared to those exposed to 4‐week IM (*p* < 0.01) (Figure 4E). *Tnfsf11* (receptor activator of NFκB ligand [RANKL]) was reduced in MSC cocultured with both 4‐week (*p* < 0.001) and 8‐week (*p* < 0.01) IM (Figure 4F). *ColIα1* (collagen type Iα1) expression in MSC cocultured with IM was not different from control cells (Figure 4G).

**Figure 5:**
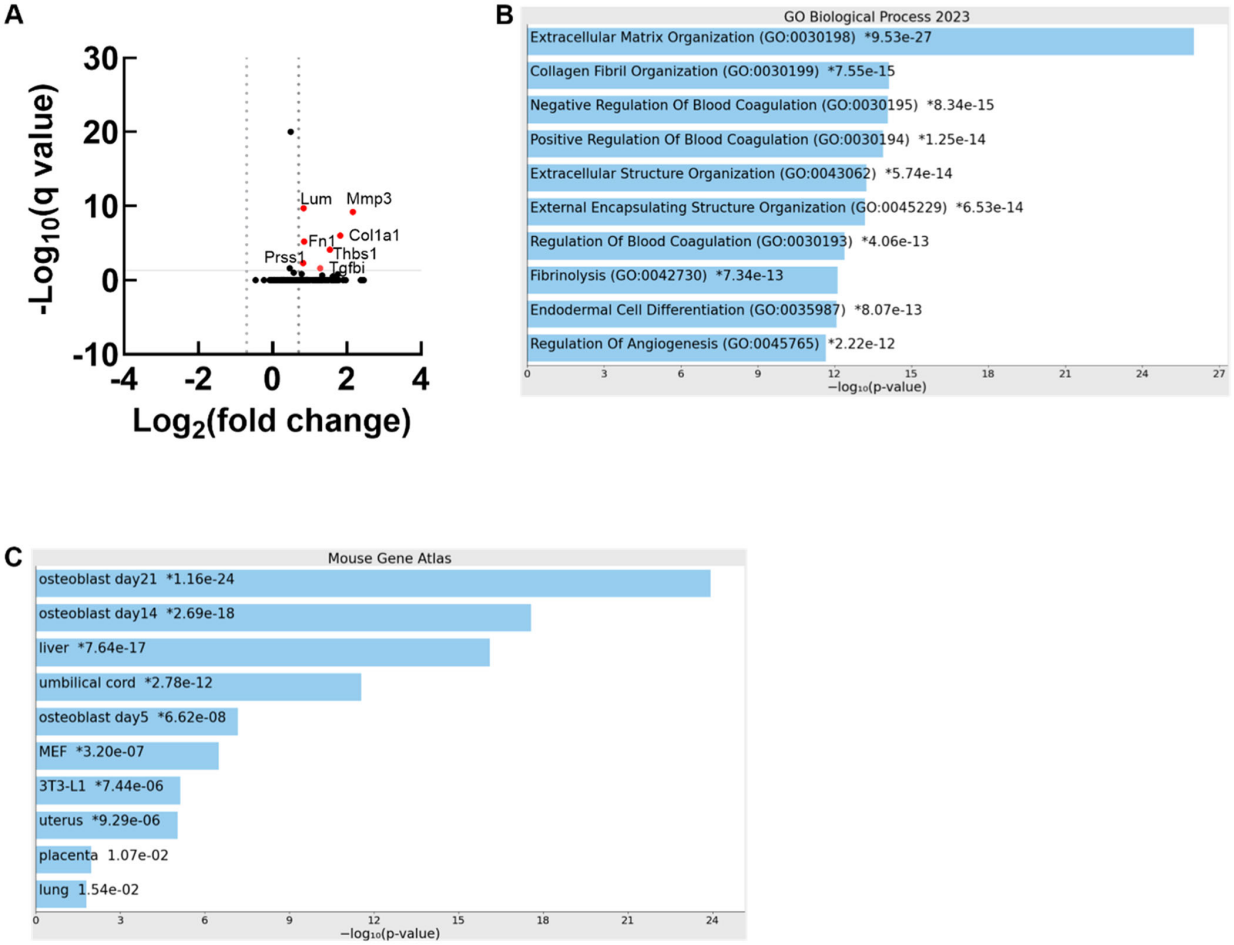
Secretomes of 4‐ and 8‐week IM have similar composition. (A) Volcano plot demonstrating time associated increases in 7 of 150 proteins secreted by IM (red dots). (B) GO Biological Process Terms enrichment analysis of the whole IM secretome. (C) Cell type enrichment analysis of the whole IM secretome (https://maayanlab.cloud/Enrichr/) IM conditioned medium from 2 to 3 different animals was combined to achieve 3 biological replicates per IM development interval for proteomics.

To unbiasedly identify secreted factors that potentially mediate IM‐induced changed in MSC gene expression, we performed proteomics analysis on serum‐free medium conditioned by untreated IMs. 150 unique secreted proteins were detected and 6 of these were significantly elevated (fold change ≥ 2 and FDR corrected *p* < 0.05) in 8‐ versus 4‐week IMs (Figure 5A and Supporting Information Table S1). 82% of the 150 proteins detected and 6 of the 7 differentially abundant proteins were also detected at the mRNA level by bulk RNA sequencing of IM allowed to develop 4 weeks in this rat model [15]. Three differentially abundant secreted proteins, TGF beta induced (TGFbi), matrix metalloproteinase 3 (MMP3), and fibronectin were more abundant at the mRNA level in IM compared to non‐union tissue [15].

Since the composition of 4‐week and 8‐week IM conditioned medium was similar, we conducted GO term analysis of the entire protein list using EnrichR. As expected, terms associated with matrix maturation were enriched (Figure 5B). Also consistent with our previous results, cell type enrichment suggested that the predominate cell types in IM express osteoblastic genes (Figure 5C).

In addition to osteoblast lineage cells, our previously published [15] bulk RNA sequencing results suggest that macrophages are present in the IM at 4‐weeks after spacer placement. Here, induction of Thbs2 gene expression in MSC by 8‐week IM was abrogated by pretreatment with clodronate, suggesting a role for IM‐resident macrophages in regulating MSC gene expression. To estimate the predominate phenotype of macrophages present in IM tissue, we compared the proteins detected here to a published RNA sequencing data set obtained from rat macrophages undergoing M1 and M2 polarization in vitro. 70 of the 150 proteins we detected in IM conditioned medium displayed significant increases in gene expression during in vitro M1 or M2 polarization (Table S2). Specifically, thirty‐five proteins detected in IM‐conditioned were enriched at the mRNA level upon M1 polarization, and twenty‐nine were enriched upon M2 polarization. The remaining five proteins were elevated upon both M1 and M2 polarization. With the caveat that the M1, M2 scheme is a simplification, this manual comparison suggests that the macrophages present in the IM were not skewed towards M1 or M2, but rather express a range of genes associated with macrophage polarization.

## 4 Discussion

Management of segmental bone defects is a significant problem for surgeons, patients, and families. MIMT is an attractive surgical procedure because it is straight forward, can be performed in relatively low resource care environments, and it performs as well as other standard surgical procedures to manage segmental bone defects (reviewed in reference [3]). Similarly, a recent systematic review and meta‐analysis of six studies (364 patients) comparing MIMT to distraction osteogenesis (DO) found similar graft to bone consolidation rates (88.2% MIMT vs. 95.7% DO), similar functional outcomes, and similar rates of later amputation. Residual angular deformity was the only significantly different outcome (9.7% MIMT vs. 1.9% DO) [21]. MIMT is also attractive because unlike DO, defect size does not correspond with healing time [8, 22].

The biological or physical contributions of the IM that forms around the cement spacer to bone consolidation after grafting are not completely clear. This is because the IM changes over time, and yet MIMT appears to be successful even when the two surgical procedures are spaced very far apart. As the IM matures into a fibrous ECM‐rich capsule, it becomes thinner [7, 23]. Elevations in secreted fibronectin, type I collagen, TSP1, and TGFβi are consistent with this phenomenon, but the thickness of the fibrous layer adjacent to the spacer did not change between 4‐ and 8‐weeks post MIMT. Similarly, DNA content and tissue weight were positively correlated at both 4 and 8 weeks of IM development suggesting the IM remained equivalently cellular for the duration of our in vivo component.

Treating IM with clodronate before coculture uncoupled the positive linear correlation between DNA content and wet weight in 8‐week but not 4‐week IM, suggesting that macrophage lineage cells may have comprised a more substantial percentage of the total IM cellular component at 8 weeks compared to 4 weeks. Taken together, our data suggest that while the IM becomes more consolidated between 4 and 8 weeks, it remains highly cellular. This distinction informs interpretation of our coculture data, and it may also indicate that animal specific attributes such as age at time of surgery may impact IM development. Direct coculture using patient‐derived IM and human osteoblast lineage cells support the notion that the IM is pro‐osteogenic and that in human patients this capability may decrease with increasing IM age [4].

Factors secreted by 4‐and 8‐week IM had differential impacts on gene expression in primary rat marrow derived MSC. Of the genes examined in MSC, alkaline phosphatase (*TNAP*) was the only one induced by 4‐week IM but not by 8‐week IM. Since the IM‐secreted proteins detected were all elevated in 8‐week versus 4‐week IM conditioned medium, one or more of these likely inhibits *TNAP*. Of the differentially abundant IM secreted factors, TSP1 is known to inhibit *TNAP* expression and osteoblast differentiation [24, 25]. On the other hand, the *Tnfrsf11b* (OPG) and *tnfsf11* (RANKL) expression patterns suggest that 8‐week IM conditioned medium is potentially antiosteoclastogenic and are counter to known impacts of TSP on these genes. Thus, TSP1 inhibits OPG and enhances RANKL in periodontal ligament fibroblasts through a p38MAP kinase dependent mechanism [26]. MMP3 is also known to inhibit MSC osteoblast differentiation [27], and TGFβi inhibits osteoblast differentiation, including *TNAP* expression via alphaVbeta3 integrin [28]. The pro‐osteogenic effects of lumican [29] were evidently not strong enough to overcome factors present in 8‐week IM conditioned medium that attenuated MSC *TNAP*. Pretreating IM with clodronate did not affect MSC *TNAP* expression, so the likely source of the factor regulating MSC *TNAP* expression is not macrophages but rather osteoblast or fibroblast lineage cells present in the IM. MSC *SPP1* expression was inhibited when cocultured with both 4 and 8‐week IM. Anti‐osteogenic impacts of TSP1, MMP3, and TGFβI are candidate mediators of this effect.

Induction of *Thbs2* by 8‐week IM was uniquely inhibited by clodronate treatment and suggests that macrophages may regulate *Thbs2* expression in MSC. Of the proteins that were significantly more abundant in 8‐week IM secretome compared to 4‐week, TSP1 and MMP3 are enriched in M1‐like macrophages, while Lum and TGFbi are enriched in M2‐like macrophages [30]. None are known to directly regulate *thsb2* expression. TSP2 is a matricellular protein that inhibits MSC proliferation and promotes osteoblast lineage progression (reviewed in reference [31]).

Like all foreign body responses, the balance between properly controlled inflammation, angiogenesis and fibrosis determines the phenotype of an IM. A robust IM vascular bed could enhance graft to bone union after the second MIMT surgery, and differences in IM vascularity may have contributed to the paracrine effects on gene expression that we document here. Wang et al have proposed utilizing TGFβ signaling antagonists to control fibrosis and thereby help maintain the IM vascular bed [32]. In their paper, they focused on extending the efficacy of an endogenous TGFβ inhibitor, fibromodulin. In their work, fibromodulin levels peaked 4 weeks after MIMT, in parallel with IM thickness and vascular density. At 6 and 8 weeks, endogenous fibromodulin decreased and Smad2/3 phosphorylation increased. Fibromodulin supplementation resulted in sustained attenuation of TGFβ signaling and longer maintenance of the vascular bed. Extracellular matrix proteins, including the antiangiogenic TGFβ target gene, TGFβI [33] were elevated in 8‐week vs 4‐week IM secretomes. Our result is consistent with increased TGFβ signaling and fibrosis in 8‐week IM compared to 4‐week IM. However, we did not detect fibromodulin in IM‐conditioned medium (Supporting Information Table S1) nor in our bulk RNAseq of 4‐week IM [15]. One major difference between these MIMT experiments is animal age: our rats were 16 weeks old, and the rats utilized by Wang et al. were 7‐weeks old. Together, the data point to the possibility that endogenous modulators of TGFβ and ECM maturation may be differentially expressed in IMs formed by different aged animals.

In conclusion, we have established a robust, reproducible coculture system for evaluating interactions between the IM and osteoblast precursor cells. Specifically our data suggest that factor(s) secreted by IM resident cells affect MSC phenotype and that the duration of IM development influences the nature of this paracrine effect. Thus, our results suggest that the IM facilitates graft to bone consolidation through paracrine mechanisms. From a translational perspective, we envision that IM pieces and excess autologous bone graft can be collected from patients undergoing the second MIMT surgery. Graft‐derived MSC and IM tissue placed into coculture will be useful for exploring mechanisms through which human IM influences bone regeneration and will permit us to determine how variables such as sex, age, and comorbidities can influence IM efficacy. Ultimately, co‐cultures established from patient‐derived IM have the potential to lead to the discovery of cellular phenotypes and secreted factors that support graft to bone consolidation after the second MIMT surgery. These could be monitored and leveraged to optimize graft to bone consolidation even in situations where surgical timing, patient history, or IM development are suboptimal.

## Author Contributions

Meredyth Bowman performed in vitro experiments, drafted the article. Gracie Sclamberg performed in vitro experiments, drafted the article. Emma Wessels assisted with surgeries, imaging, animal care. Kyle Cragg assisted with surgeries, imaging, animal care. Alexis Donneys was the surgeon of record for all procedures. Kurt D. Hankenson contributed to article revision and interpretation of data. Mark E. Hake gave input on the preclinical model of MIMT. Andrea I. Alford conceived the study, performed experiments, edited the manuscript. All authors have read and approved the final submitted article.

## Conflicts of Interest

The authors declare no conflicts of interest.

## Supporting information

Supporting information.
